# Supplementary material for: The roles and signalling pathways of lncMALAT1 in coronary artery disease: A protocol for systematic review of in vivo and in vitro studies
Source: PLoS One. 2025 May 5;20(5):e0322550. doi: 10.1371/journal.pone.0322550 (PMC12052108; doi:10.1371/journal.pone.0322550)
Supplement: S1 Table — (DOCX) [file pone.0322550.s001.docx]

| Supporting Information S1Table 1: Eligibility criteria based on the Population, Intervention, Comparison, Outcome and Study framework | | |
| --- | --- | --- |
|  | **Inclusion criteria** | **Exclusion criteria** |
| **Population** | Animal study: primary CAD animal model (established with coronary artery in acute and chronic operation (not limited to coronary artery ligation, ischemia/reperfusion, cryo-/electrical injury, micro-embolism, pharmacological induction, genetic models) of any type, given at any time and diet condition, all sex, all age, and all species.  In vitro study: any cardiomyocyte cell line (all human and animal) primary hypoxia model (established with low oxygen concentration or cobalt chloride agent ) in any level, given at any time, concentration, frequency, and duration. | Animal studies: CAD model that involved aorta stenosis, heart failure and cardiomyopathy.  In vitro study: cardiomyocytes derived from the induced stem cells, and genetically modified cardiomyocytes. |
| **Intervention/ exposure** | Animal study:  i. Gene therapy: any viral vector (e.g. lentivirus, recombinant adenovirus) in any dosing, given at any time and frequency of dosing.  ii. Cells: any types of cells (e.g. cardiac cells, stem cell including bone marrow mesenchymal, adipose mesenchymal, umbilical cord mesenchymal) in any dosing, given at any time and frequency of dosing.  iii. Drugs: CAD drugs in any dosing, given at any time and frequency of dosing.  iv. Device: invasive (coronary stent or scaffolds, coronary balloon catheter) in any material, non-invasive (hyperbaric oxygen) in any oxygen concentration, will be given at any time, duration, and frequency.  In vitro study:  i. Gene therapy: any viral vector (e.g. lentivirus, recombinant adenovirus) in any concentration, given at any time and frequency of concentration.  ii. Cells: cell-derived extracellular vesicles (e.g. exosomes, micro-vesicles or both) in any concentration, given at any time and frequency of concentration. No restriction on method of isolation and purification.  iii. Drugs/agents: in any concentration, given at any time and frequency of concentration. | Animal study: animal model that did not report signalling pathways and vascular changes.  In vitro study: cell culture model that did not report signalling pathways and biological changes. |
| **Comparator** | For animal study, at least any two of these three groups will be included:  i. Negative control group (placebo, vehicle vector)  ii. Positive control group (drugs/agents, cells, device)  iii. Healthy group  with or without  iv. Sham group  For in vitro study, at least any two of these three groups will be included:  i. Negative control group (placebo, viral vector)  ii. Positive control group (drugs/agents)  iii. Healthy group | Animal study: Studies without comparator or control groups necessary for meaningful interpretation of experimental outcomes related to lnc-MALAT1 in CAD.  In vitro study: Studies without control conditions (normal oxygen concentration of standard cell culturing protocol (37℃, CO₂ 5%) or those failing to implement controls essential for accurate assessment of the role and signaling pathway of lnc-MALAT1 in hypoxia progression. |
| **Primary** | | |
| **Outcome** | Animal study: Klotho/FGF23 and Wnt/β-catenin and ERK/MAPK pathways.  In vitro study: Klotho/FGF23 and Wnt/β-catenin and ERK/MAPK pathways. | |
| **Secondary Outcome** | Animal study: Other signalling pathways and vascular change (proliferation, migration, lumen formation, apoptosis).  In vitro study: Other signalling pathways and biological changes (proliferation, migration, lumen formation, apoptosis). | |
| **Study type** | Original research articles in English language.  Animal studies: Controlled experimental studies utilizing CAD animal model. Randomized controlled studies or other controlled experimental design.  In vitro studies: Controlled experimental studies utilizing hypoxia conditions. Other controlled experimental design. | Full texts are not available  Reviews  Systematic reviews  Meta-analyses  Conference abstracts and Non-English language  Letter to editors  Book  Editorial comments  Abstract  Conference proceedings. |
| PICOS: Population, Intervention, Comparison, Outcome and Study | | |
