# Supplementary material for: The roles and signalling pathways of lncMALAT1 in coronary artery disease: A protocol for systematic review of in vivo and in vitro studies
Source: PLoS One. 2025 May 5;20(5):e0322550. doi: 10.1371/journal.pone.0322550 (PMC12052108; doi:10.1371/journal.pone.0322550)

| Table 1: Search strategy in Pubmed, Web of Science and Scopus databases | |
| --- | --- |
| **Search#** | **Search items** |
| #1 | MALAT1 (All Fields) |
| #2 | lncMALAT1 (All Fields) |
| #3 | #1 or #2 |
| #4 | “coronary artery disease” |
| #5 | “atherosclerosis” |
| #6 | “myocardial infarction” |
| #7 | “unstable angina” |
| #8 | “myocardial bridge” |
| #9 | “myocardial ischemia” |
| #10 | #4 or #5 or #6 or #7 or #8 or #9 |
| #11 | #3 AND #10 |

**Pubmed**

https://pubmed.ncbi.nlm.nih.gov/


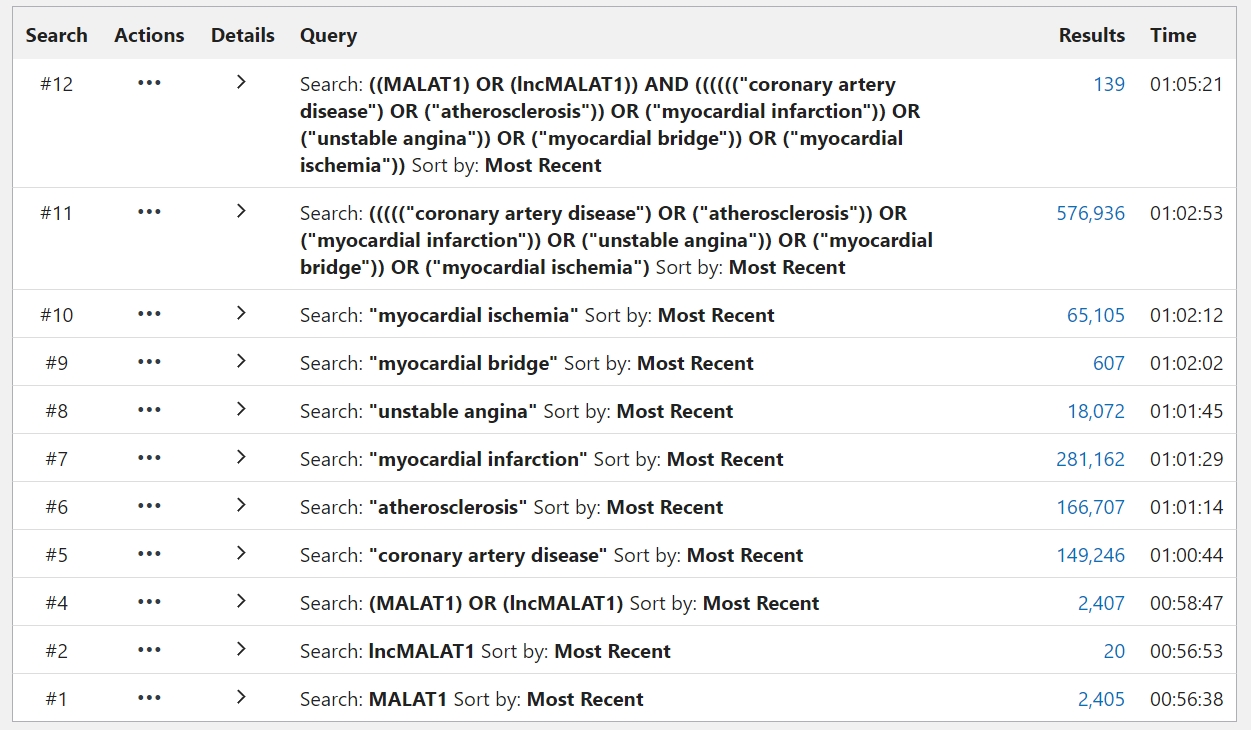


**Scopus**

https://www-scopus-com.eresourcesptsl.ukm.remotexs.co/search/form.uri?display=basic#basic


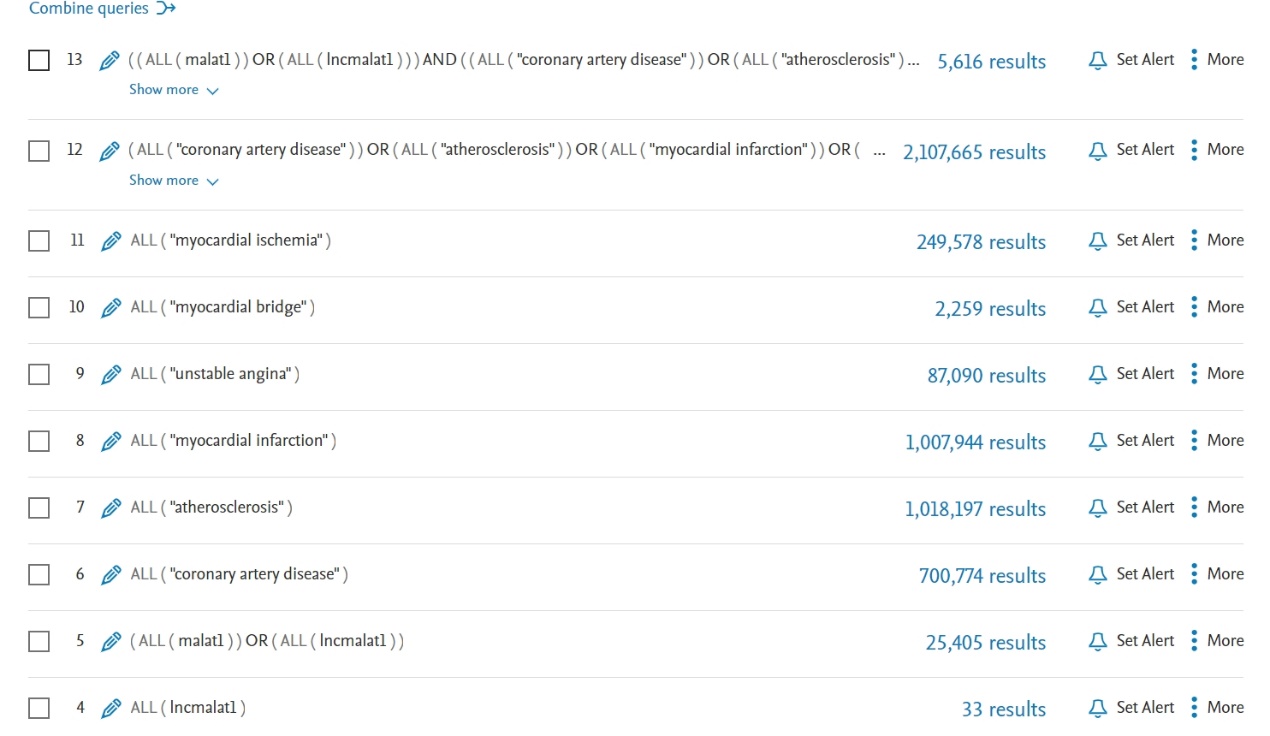


**Web of Science**

https://www-webofscience-com.eresourcesptsl.ukm.remotexs.co/wos/woscc/basic-search


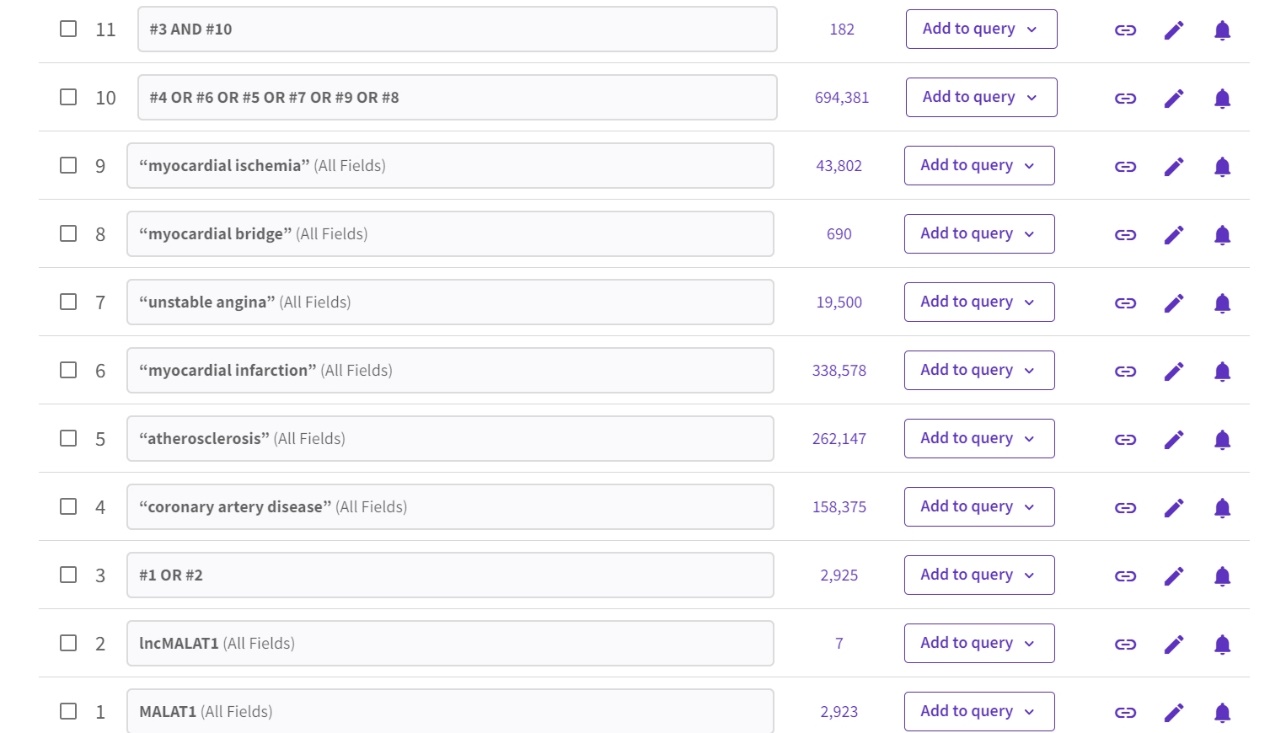

Supplement: S2 Table — (DOCX) [file pone.0322550.s002.docx]
