# Supplementary material for: The roles and signalling pathways of lncMALAT1 in coronary artery disease: A protocol for systematic review of in vivo and in vitro studies
Source: PLoS One. 2025 May 5;20(5):e0322550. doi: 10.1371/journal.pone.0322550 (PMC12052108; doi:10.1371/journal.pone.0322550)
Supplement: Attachment — Submitted filename: Response to Reviewers.docx [file pone.0322550.s006.docx]

| **Reviewer Comments** | **Response** | **Revised Location in Manuscript (file name: Revised Manuscript with Track Changes)** |
| --- | --- | --- |
| **Editor** | | |
| PLOS ONE  Journal Requirements:  Please review your reference list to ensure that it is complete and correct. If you have cited papers that have been retracted, please include the rationale for doing so in the manuscript text, or remove these references and replace them with relevant current references. Any changes to the reference list should be mentioned in the rebuttal letter that accompanies your revised manuscript. If you need to cite a retracted article, indicate the article’s retracted status in the References list and also include a citation and full reference for the retraction notice.  [Note: HTML markup is below. Please do not edit.] | Dear ***PLOS ONE*** Editors,  Greetings.  We appreciate your suggestion regarding the completeness and accuracy of the reference list. After thorough crosscheck for each citation with trusted databases, including PubMed and Web of Science since receiving the Journal's requirement, we have verified that there are none of the retracted articles being cited in our manuscript. Moreover, we have also ensured that all references in our manuscript are relevant to the topic. Therefore, no changes to the reference list are necessary. If there are any further details or adjustments, please feel free to let us know. |  |
|  |  |  |
| **Reviewer #3** | | |
| Overall: | | |
| The revised manuscript has addressed all the points raised in the initial review. The improvements made are satisfactory, and the manuscript is now acceptable for publication. | Dear Prof. Sareh Bakhshandeh Bavarsad  Greetings.  We sincerely thanks for your time, effort, and thoughtful consideration in accepting our revised manuscript for publication. We are pleased to hear that our previous revisions have addressed all the concerns raised in the previous review and that the improvements have met your expectations. We greatly appreciate the detailed and comprehensive review process, as well as the constructive feedback provided. |  |
|  |  |  |
| **Reviewer #4** | | |
| Overall: | | |
| Thanks for the opportunity to review this manuscript. I think everything is fine. However, I may have a few concerns: | Dear Prof.,  Greetings.  We deeply appreciate your thorough contribution of our manuscript, and comprehensive review process, as well as the valuable comments provided. Your comments are valuable for further improving the manuscript. We have carefully considered each item and have made the following responses and revisions to address your concerns. |  |
| Comments: | | |
| 1. I think putting a section named "results" in the abstract is somehow redundant for a protocol study. Consider removing it. | We appreciate your thoughtful comment regarding the inclusion of a "Results" section in the abstract. We understand the concern that protocol manuscript not typically to present results in the abstract.  Therefore, we agree with your point that the inclusion of this section may be unnecessary and potentially redundant in the context of a protocol manuscript. Next, we removed the “Results” section in the abstract of the revised manuscript, ensuring that it better matches the format and expectations of a protocol study. | Page 2, line 46-49 |
| 2. Please provide full strategy for searching each database as a table in your manuscript. | We fully appreciate your valuable comment regarding the importance of providing a clear and transparent strategy for database searches in a systematic review protocol, as it contributes to ensure reproducibility for any site performing this process.  However, we have included the full search string in the revised manuscript to address your concern, even though the full strategy for searching each database is present in Table 1 and Supporting information S2 of the current manuscript. This detailed full strategy information on the search terms and relevant parameters for each database, ensuring that the methodology is fully transparent and easily replicable. | Page 7, line 188-191 |
| 3. In your PRISMA figure, please state the full precise exclusion reasons for the full-text reviewing stage. | We greatly appreciate your suggestion regarding the PRISMA flow diagram, which has further strengthened our protocol. We recognize the importance of providing clear and precise reasons for exclusions during the full-text review stage, as it enhances the transparency and rigor of the systematic review process.  Moreover, we have updated the PRISMA diagram to include the full and precise reasons for exclusions at the full-text review stage to address your concern. These reasons are clearly mentioned, which may improve the clarity and completeness of the manuscript. | Page 10, Figure 1 PRISMA flow diagram for systematic reviews |
| 4. First paragraph of the introduction is a bit too long. Consider summarising it. | We greatly appreciate your constructive comment. We agree with your point that the first paragraph of the introduction was somewhat lengthy and could benefit from greater conciseness to enhance readability.  To address your concern, we have revised the first paragraph, summarizing the key points more concisely while retaining the essential information. We believe the revision improves the readability of the introduction and ensures a clearer presentation of our objectives. | Page 3-4, line 61-79 |
