# Supplementary material for: The roles and signalling pathways of lncMALAT1 in coronary artery disease: A protocol for systematic review of in vivo and in vitro studies
Source: PLoS One. 2025 May 5;20(5):e0322550. doi: 10.1371/journal.pone.0322550 (PMC12052108; doi:10.1371/journal.pone.0322550)
Supplement: Attachment — Submitted filename: Response_to_Reviewers_auresp_3.docx [file pone.0322550.s007.docx]

| **R1** | | |
| --- | --- | --- |
| **Reviewer Comments** | **Response** | **Revised Location in Manuscript (file name: Revised Manuscript with Track Changes)** |
| **Reviewer #1: Mojtaba Mehrabanian** | | |
| Overall: | | |
| This protocol has a strong foundation for a systematic review. The study has the potential to make a valuable contribution to the field of cardiovascular research, particularly in understanding the role of lncMALAT1 in CAD. With the recommended additions, this study has the potential to be a valuable contribution to the understanding of lncMALAT1 in CAD | Dear Prof. Mojtaba Mehrabanian,  Thanks for your comprehensive and valuable comments. We deeply appreciate to your vulnerable contributions to our study protocol article. For your concern, we would like to try our best do the revision and explanation to make the manuscript more readable. |  |
| Comments: | | |
| 1. Expand the search strategy to include Embase or CINAHL for a more comprehensive literature search. | We appreciate the suggestion to expand our search strategy to include Embase or CINAHL. After careful consideration and discussion, we have decided to maintain the current search strategy using PubMed, Web of Science, and Scopus for the following reasons:  (1) Wide coverage: PubMed, Web of Science, and Scopus are among the largest and most comprehensive databases for biomedical literature. They collectively cover a majority of peer-reviewed journals in the field of cardiovascular disease and molecular biology.  (2) Specific research area: We focus on a specific molecular pathway ---lncMALAT1 in CAD. Compared to the current databases, the CINAHL is insufficient in retrieve literatures that related to molecular biology and cardiovascular disease. EMBASE is very focus on technique-based aspect.  (3) Avoiding redundancy: There is possible that significant overlap between the content of Embase/CINAHL and the chosen databases. Moreover, there may lead to a number of duplicate entries without increasing valuable and relevant studies.  We believe this search strategy will collect the majority of relevant literature while maintaining a focused and efficient review process. However, we acknowledge that this decision maybe a limitation of our study, and we will have a statement in the limitations section. We appreciate your understanding and available discussion if you have any additional concerns about our search strategy. |  |
| 2. Provide a more detailed plan for how data will be extracted, managed, and synthesized. This should include specific tools or software that will be used and methods for resolving discrepancies between reviewers. | We acknowledge the need for a more detailed plan in the section of data extraction and synthesis. We have expanded description of this section to the revised manuscript.  We would like to revise this section as you suggested:  A comprehensive, standardized data extraction form will be developed using Microsoft Excel and piloted for variable extraction by two independent reviewers (JZ and NFJ). The identified citations and extracted data will be regularly backed up to prevent data loss. Demographic data will include the first author’s name, year of publication, country, study design, and experimental study used to form an initial development. Two reviewers (JZ and NFJ) will pilot the form on a random sample of 20% studies, which representing diverse study designs, that meet the inclusion criteria. The reviewers will refine the form accordingly while identified by any difficulty after discussion. The revised form will be tested on an additional 10% studies to address all concerns. The third independent reviewer (AFML) will provide any additional revision after reviewing the piloted form before finalization.  Duplicated studies will be excluded. Any significant differences in data extraction will be resolved through discussion between the reviewers. If necessary, a third reviewer (AFML) will be consulted. To ensure data quality and consistency, a random sample of 30% of the included studies will be cross-checked by a third reviewer (AFML). If significant differences are found, a full review of all extracted data will be conducted.  **Data synthesis**  For data synthesis, we will employ a narrative synthesis approach. All extracted data from eligible studies will be exactly recorded into Microsoft Excel with sufficient information by dividing into animal model and in vitro model two parts. The synthesized data will be presented in tabular or graphical form. | Page 10-11, line 238-264; Page 12, 282-286. |
| 3. Include a broader discussion about potential biases and the steps that will be taken to mitigate them. This should cover publication bias, selection bias, and other biases related to the included studies. | We appreciate your opinion in addressing potential biases more comprehensively. We have expanded description of this section to the revised manuscript.  We would like to revise this section as you suggested:  **Quality assessment and bias assessment**  To minimize selection bias, we will employ independent screening of titles, abstracts, and full texts. Any disagreements will be resolved through discussion or consultation by a fourth reviewer (NFR). Considering study limitations, incomplete outcome, imprecision, and publication bias, any discrepancies or disagreements, a fourth reviewer (NFR) will be consulted if possible. | Page 10, line 266; 276-280. |
|  |  |  |
| **Reviewer #2: Amin Salehi-Abargouei** | | |
| Overall: | | |
| A manuscript trying to explain the protocol for a systematic review and meta-analysis of in vitro and animal studies examining the roles and signaling pathways of lncMALAT1 in coronary artery disease. There are a number of concerns that should be solved before its publication: | Dear Prof. Amin Salehi-Abargouei,  Greetings.  We deeply appreciate your thorough contribution of our manuscript and the valuable comments provided. We have carefully considered each item and have made the following responses and revisions to address your concerns. |  |
| Comments: | | |
| 1- The PICOS should be explained both for animal and in vitro studies. | We appreciate your concern about the explanation both for animal and in vitro studies. We have explained the information from line 141-185 and this information has been included in the file name “Supporting Information S1 Table 1: Eligibility criteria based on the Population, Intervention, Comparison, Outcome and Study framework”. We would like to clarify that we have prepared a supporting information table. This information is provided in a tabular way, which outlines the PICOS for both animal and in vitro studies separately. | Page 5, line 137-138; Page 7, line 184-185.  Supporting Information S1 |
| 2- What is the primary outcome in the currents study. It should be explained. What are the secondary outcome? | We would like to present primary outcome for both animal and in vitro studies with the description of Klotho/FGF23 and Wnt/β-catenin and ERK/MAPK pathways. Similarly, the second outcome will be present for both animal and in vitro studies with the description of other signalling pathways from the primary outcome and vascular change (proliferation, migration, lumen formation, apoptosis). We present the information in “Supporting Information S1 Table 1” to avoid any overlap with the main manuscript. | Page 7, line 170-176.  Supporting Information S1 |
| 3- A wide range of interventions are also selected for this study. | We appreciate your observation about the range of interventions in our study. However, we consider that the range of interventions is appropriate and necessary for the comprehensive evaluation to the aim objectives. We would like to have some following explanation to address your concerns:  CAD in vivo models: The interventions in CAD animal models are designed to simulate the myocardial ischemic hypoxia state characteristic of coronary artery disease (CAD). These approaches are established and already been published to initiate hypoxia condition that led to CAD. These models contribute us to observe the role of lncMALAT1 in a complex physiological environment that closely mimics human CAD.  In vitro models: The interventions in cell models aim to present the hypoxic state at a cellular level. While these models may have limitations, they allow for more precise observation of molecular pathways involving lncMALAT1.  By including both in vivo and in vitro models, we acknowledge that while these models may have similar pathophysiological environments, they might reveal different mechanisms due to the inherent limitations of in vitro systems.  This dual approach will benefit us to:   [1] Capture a clearer role of lncMALAT1 in CAD; [2] Cross-validate findings between different model systems; [3] Identify potential discrepancies between in vivo and in vitro results, which could highlight CAD areas for future research.   We would like to believe that the comprehensive evaluation of interventions from both in vivo and in vitro perspectives contribute to provide more convincing and credible evidence regarding the roles and signaling pathways of lncMALAT1 in CAD. This approach aligns with the complex nature of CAD and the multi-roles that lncMALAT1 may play in its pathophysiology. | Page 6-7, line 141-168; Supporting Information S1 |
| 4- The authors also aim to explore the mechanisms. The mechanisms are not the aim of systematic reviews and meta-analyses. As these studies are designed to provide evidence on effects and associations. The topic seems to be too wide for a systematic review. | We appreciate your opinion to the primary purpose of systematic reviews. We would like to clarify our objectives and approach:  We indeed primarily focus on synthesizing evidence on the effects and associations of lncMALAT1 in CAD, rather than exploring mechanisms. The aim of our study is to provide a protocol for systematically review and synthesize the current evidence on the role of lncMALAT1 in CAD from both in vivo and in vitro studies.  Specifically:  (1) Summarize the observed effects of lncMALAT1 expression changes in CAD models.  (2) Synthesize evidence on the associations between lncMALAT1 and CAD-related primary and secondary outcomes.  (3) Analyze and descript data on signaling pathways that are consistently reported in related studies to lncMALAT1 and CAD.  While we will report on signaling pathways mentioned in the included studies, our objective is not to explore or elucidate new mechanisms. Instead, we aim to provide a comprehensive overview of the current evidence regarding the role of lncMALAT1 in CAD.  This approach aligns with the primary objective of systematic review, which is to summarize and synthesize existing evidence. By focusing effects and associations on in vivo and in vitro models rather than mechanisms, we would like to consider our review will provide valuable insights into the current understanding of lncMALAT1 in CAD. | Page 2, line 37-45; Page 5, line 116-125; Page 12, line 282-297. |
| 5- It seems that the search is already completed (Based on abstract). This is while I think the strategy is not complete. As outcome CVD markers of interest are not included in the search strategy. | We appreciate your concern about the search strategy. We would like to clarity our study objective is to systematically review and synthesize the in vivo and in vitro studies on the role and signaling pathways of lncMALAT1 in CAD. Considering the broad nature of this objective and the potential for lncMALAT1 to be involved in various aspects of CAD, we have designed a comprehensive search strategy. We would like to consider that narrowing the search scope may cause the occasionally omission of relevant literature. Our current strategy aims to capture a wide range of studies that investigate lncMALAT1 in the context of CAD, including those that may not explicitly mention specific CAD markers in titles or abstracts.  We understand your concern about the inclusion of interest CAD markers in the search strategy. However, we would like to consider that pre-defining these markers might introduce bias by potentially excluding studies that investigate novel or less common markers, hence, affecting our secondary outcome. The description and analysis of the interest CAD markers will be presented after the subsequent study is completed. This approach allows us to report on all relevant markers that emerge from our comprehensive search strategy.  To address any concerns about the management of the search results:  We will employ a rigorous screening process to ensure that just closely relevant studies are included. Our detailed inclusion and exclusion criteria will guide this screening process, ensuring directly address our focused research questions. Therefore, we would like to believe this comprehensive approach will provide the most complete and unbiased representation of the current literature on lncMALAT1 in CAD. |  |
| 6- Is any meta-analysis planned for this study? If yes, it should be explained. | We appreciate your inquiry about our plans for meta-analysis. We would like to clarify that we will not conduct a meta-analysis as part of this systematic review.  Considering the diversity of in vivo and in vitro models, interventions, and outcomes in studies of lncMALAT1 in CAD, we anticipate significant heterogeneity among the included studies. This heterogeneity may make a meaningful challenge to quantitative synthesis. Our primary objective is to provide a comprehensive overview of the current evidence on the role of lncMALAT1 in CAD. As this is a relatively new area of investigation, many studies may be with various methodologies and outcome measures. We would like to consider that a narrative synthesis will be more appropriate for capturing the complexities of the various studies in the field of CAD. | Page 1, line 4-5; Page 2, line 37, 47; Page 4, line 104-105; Page 5, line 116-125; Page 12, line 288-290. |
| 7- What would be the method for the synthesis of results? | We will employ a narrative synthesis approach. The narrative synthesis will be structured around our research questions, with data grouped by study design, experimental model, and outcomes. The method for the synthesis of results was revised in manuscript. The descriptive analysis will be used to report the data. | Page 11, line 282-286. |
| 8- How the quality of in vitro studies is going to be assessed? | We appreciate your concern about the quality assessment of in vitro studies. We would like to clarify our approach to quality assessment for both animal and in vitro studies:  As presented in our manuscript, we will employ the SYRCLE's risk of bias tool for quality assessment of all included studies, including in vitro studies. This tool was chosen for its comprehensive approach and adapt to different types of experimental studies. Our quality assessment criteria including:  i) were inclusion/ exclusion criteria reported;  ii) was the search adequate;  iii) were the included studies synthesized;  iv) was the quality of the included studies assessed;  v) are sufficient details about the individual included studies presented.  We recognize the importance of randomization and heterogeneity in both animal and in vitro studies to provide a more accurate reflection of reality. While the SYRCLE's tool was originally developed for animal studies, we believe its core principles are applicable to in vitro studies as well, with appropriate adaptations.  To ensure a rigorous quality assessment process:  We will require studies to meet at least four of the five criteria to be included in our review, ensuring a high standard of quality across all included studies.  The quality assessment will be conducted independently by three reviewers (JZ, NFJ, and AFML) to minimize bias and ensure consistency.  For the specifically of in vitro studies, we will pay particular attention to the example aspects:  1) Appropriate use of controls;  2) Replication of experiments;  3) Reporting of cell line authentication;  4) Description of culture conditions and treatments;  5) We will employ a random effect models in our study, which provides a high level of evidence as noted in our manuscript.  We would like to consider this comprehensive quality assessment approach to both animal and in vitro studies contribute to the high-quality evidence of systematic review. | Page 11-12, line 266 - 280. |
|  |  |  |
| **Reviewer #3** | | |
| **Comments:** | | |
|  | Dear Prof,  Greetings.  We deeply appreciate your thorough contribution of our manuscript and the valuable comments provided. We have carefully considered each item and have made the following responses and revisions to address your concerns. |  |
| 1. Background |  |  |
| Could be enhanced by including a detailed discussion of the gaps in current knowledge that this systematic review aims to fill. | We appreciate your suggestion regarding the enhancement of background section. We would like to consider that the current background is concise but highlight the essential topics and context for our systematic review effectively.  1) It clearly presents CAD as a major global health issue, setting the stage for the significance of our study.  2) Currently, the association between high expression levels of lncMALAT1 and increased risk of CAD is still unknown clearly.  3) The current background explicitly states the aim of our systematic review: to evaluate the role and mechanism of lncMALAT1 that may contributed to CAD based on animal and in vitro studies.  4) It will descript some interest specific signaling pathways such as (Klotho/FGF23, Wnt/β-catenin, ERK/MAPK), as well as other potential pathways, providing a clear scope for our review.  5) The current background also mentioned the potential vascular changes (proliferation, migration, lumen formation, and apoptosis), further present the relevant outcomes.  We would like to consider this concise and comprehensive background effectively sets specific aims for our systematic review protocol. It provides readers with the necessary context to understand the importance of our study effectively without excessive detail. | Page 2, line 27-35. |
| 2. Introduction: |  |  |
| It should be expanded to include more detailed epidemiological data and recent findings related to lncMALAT1(for example: Include diagrams or figures, if possible, to visually represent these complex interactions and Biological Mechanisms). | We appreciate your suggestion to expand the introduction to include more detailed epidemiological data and recent findings related to lncMALAT1, including visual representations of complex interactions and biological mechanisms.  We would like to clarify the scope and intent of our current manuscript:  We primarily aim to present a protocol for a systematic review of lncMALAT1 related to CAD in animal models and in vitro models. Therefore, the introduction is intentionally focused on providing the essential context for understanding the need for this review. In fact, the detailed exploration of the latest findings related to lncMALAT1 and CAD is the key objective of our systematic review. We would like to consider it would be premature to present this information in the protocol stage, as doing so might introduce bias into our review process. We plan to present a comprehensive analysis of the latest findings of complex interactions and biological mechanisms, in the full systematic review article that will follow this protocol. By reserving the detailed analysis for the full review article, we maybe ensure to present the latest and comprehensive information, synthesized from all the studies included in our study. | Page 3-5, line 61-114. |
| 3. Inclusion and exclusion criteria |  |  |
| More specific inclusion and exclusion criteria (e.g., language restrictions, types of in vitro and in vivo models) should be detailed in text. Criteria for excluding studies based on quality or other factors should be stated. | We appreciate your suggestion to provide more specific inclusion and exclusion criteria in the text. We would like to clarify our approach to presenting this information. We have prepared a detailed "Supporting Information S1 Table 1" that comprehensively focus on PICOS (Population, Intervention, Comparator, Outcomes, Study design) framework, including specific inclusion and exclusion criteria for both animal and in vitro studies. To maintain the conciseness of the main manuscript and avoid unnecessary repetition, we would like to present the detailed criteria in the supporting information rather than in the main manuscript. While the detailed criteria are presented in the supporting information, we will review the main manuscript to ensure that suggested inclusion and exclusion that you mentioned. | Page 5-6, line 127-137;  Supporting Information S1. |
| 4. Study intervention and comparators |  |  |
| Comparators  It is not clear if all studies must include at least two groups or if some flexibility is allowed. This should be explicitly stated. | We appreciate your comment regarding the clarity of comparators. We would like to draw your attention to the section in the manuscript that address this concern:  For animal studies: We have explicitly stated that "at least any two of these three groups will be included: This section clearly indicates that all included studies must have at least two groups for comparison.  For in vitro studies:  Similarly, we have specified that "at least any two of these three groups will be included: See Page 6 Line 162-168.  These criteria ensure that all included studies will have appropriate comparators, contributing to meaningful interpretation of results. We would like to consider that these details could sufficiently address your concern about the clarity of comparators in our study protocol. | Page 6-7, line 141-168;  Supporting Information S1. |
| Exclusion Criteria in (Animal study)  The rationale for these exclusions should be more explicitly justified. Explain why the absence of these elements precludes meaningful interpretation of the results. | We appreciate your suggestion to more explicitly justify the rationale for our exclusion criteria in animal studies. We would like to draw your attention to the details we have provided in the manuscript and present further response:  Exclusion Criteria in (Animal study):  We have explicitly stated that "CAD models that involved aorta stenosis, heart failure and cardiomyopathy will be excluded."  Rationale for Exclusion:  Although not explicitly stated in the manuscript for reasons of conciseness, the rationale for these exclusions were based on our focus on models of primary CAD and common knowledge about the listed diseases.  Aorta stenosis: While this condition can lead to CAD, it represents a different primary pathology and may introduce confounding factors when studying the specific role of lncMALAT1 in CAD.  Heart failure: Although often a consequence of CAD, heart failure can have multiple etiologies and may involve different molecular mechanisms. Including heart failure models could potentially obscure the specific effects of lncMALAT1 in CAD.  Cardiomyopathy: This represents a distinct group of cardiomyocyte diseases that may have genetic or other reasons unrelated to CAD, and thus falls outside the scope of our study.  By focusing on primary CAD models, we would like aim to ensure that the studies included in our review directly address the role of lncMALAT1 in the pathophysiology of CAD, without the confounding effects of other cardiac pathologies. | Page 5, line 132-133;  Supporting Information S1. |
| In vitro study  Similar to the animal study section, the readability could be improved by using bullet points or subheadings. | We appreciate your suggestion to improve the readability of the in vitro study section by using bullet points or subheadings. We would like to clarify our approach:  (1) The paragraphs in the main manuscript are intentionally presented in a concise format to maintain the overall flow and readability of the manuscript. This approach allows readers to quickly grasp the key points without being overwhelmed by detailed lists.  (2) As your suggestion, we have indeed presented the detailed criteria for in vitro studies in a more structured format in our Supporting Information S1 Table 1. This table uses bullet points and subheadings to clearly present the various aspects of inclusion and exclusion criteria for animal and in vitro studies.  (3) We would like to consider this approach presents an optimal balance between providing a concise overview in the main manuscript and offering detailed with easily understandable information in the supporting documents.  (4) Readers can easily refer to the Supporting Information S1 Table 1 if require more detailed information. Supporting Information provides a comprehensive and structured presentation of our criteria.  (5) This approach aligns with PLOS ONE's guidelines for manuscript structure, which encourage the use of supporting information for detailed methodological aspects. | Page 5-6, line 134-137;  Supporting Information S1. |
| Outcome measures  While the identified pathways are relevant, the rationale for choosing these particular pathways should be provided. Explain why these pathways are of primary interest and how they relate to the research question.  The term "other signaling pathways" is vague. It would be beneficial to specify which other pathways are considered relevant or to outline criteria for identifying additional pathways. Furthermore, provide more context on how these secondary outcomes contribute to understanding the role of lncMALAT1 in CAD.  The rationale for excluding efficacy measures should be clearly stated. Explain why efficacy is not considered within the scope of this review and how focusing on mechanistic outcomes will provide more valuable insights. | We appreciate your attention to this important aspect of our protocol. We would like to revise the section of outcome measures as you suggested. These pathways have been chosen as primary outcomes due to play roles in cardiovascular pathophysiology and potential interaction with MALAT1. The secondary outcome will be the identification of other signalling pathways and vascular/biological changes (proliferation, migration, lumen formation, apoptosis), as these processes are crucial in the development and progression of CAD.  In further, we would like to consider the criteria for including additional pathways will be based on their frequency of appearance in the reviewed studies and their demonstrated or hypothesized relationship to lncMALAT1 and CAD pathogenesis.  Moreover, we would like to consider efficacy outcome measures, such as changes in clinical endpoints or biomarkers, will be excluded from this review. This opinion is based on our focus on elucidating the mechanistic role of lncMALAT1 in CAD, rather than its potential therapeutic efficacy. By focusing on signaling pathways and cellular processes, we aim to provide a comprehensive understanding of how lncMALAT1 contributes to the pathophysiology of CAD, which we consider will offer more valuable insights for future research and potential therapeutic target identification. | Page 7, line 171-176. |
| Search strategy and sources  The search strategy could benefit from a more detailed explanation of how MeSH terms and keywords were selected. Providing an example of the search strings used in each database (beyond just Scopus) in the main text would improve transparency and reproducibility. | We appreciate your suggestion to provide a more detailed explanation of how MeSH terms and keywords were selected, and to include example search strings for each database in the main text. We would like to clarify our approach:  (1) We have provided a detailed information of search strategy, including MeSH terms, keywords, and complete search strings for each database (PubMed, Web of Science, and Scopus) in the Supporting Information S2 Table 1: Search strategy in PubMed, Web of Science and Scopus databases.  (2) We would like to put this detailed information in the supporting information to maintain the readability, conciseness, and focus of the main manuscript while still improve transparency and reproducibility of the search strategy.  (3) By placing the full search strings in the supporting information, we would like to consider that researchers who wish to replicate or build upon our work have access to the exact search strategy presented, while keeping the main text concise and focused. | Page 7-8, line 187-194; Page 9, Table 1;  Supporting Information S2. |
| Study selection  It would be more logical to state that duplicates will be removed after the search, as duplicates are identified once all studies have been retrieved. Consider rephrasing for clarity. | We appreciate your attention to detail regarding the study selection process. We would like to revise this section as you suggested: After conducting the search across all specified databases, one researcher (JZ) will import the retrieved studies into EndNote™ 20. Any duplicates will be removed by the duplicated identification feature of EndNote™ 20 before proceeding with the screening of potential studies. | Page 8, line 196-200. |
| To ensure the transparency of the screening process, the justification for exclusion (for example, wrong study design, wrong population, wrong exposure, wrong comparator, wrong result, etc.) should be mentioned in the study in the study selection section. | We appreciate your attention to this important aspect of our protocol. We would like to revise this section as you suggested: To ensure the transparency of the screening process, the justification for exclusion (wrong study design, wrong population, wrong language, wrong comparator, wrong outcome, etc.) will be marked in Rayyan. | Page 8, line 203-205. |
| Describe how discrepancies will be resolved (e.g., through discussion, voting, or involving a third party). This adds transparency to the process. | We appreciate your attention to this important aspect of our protocol. We would like to revise this section as you suggested: Researchers will meet upon completion to compare results. Any discrepancies will be resolved with the other researchers (AFML and NFR) through discussion or voting, which contribute to improve transparency in this process. | Page 9, line 212-213. |
| 5. Data management and extraction |  |  |
| The protocol should describe the piloting process in more detail. For example, explain how the form will be tested and refined based on feedback from the pilot phase.  Ensure that the distinction between included and excluded data is clear. The phrase "the data that will be excluded" seems to be a typographical error; it should be "the data that will be included." | We appreciate your attention to this important aspect of our protocol. We would like to revise this section as you suggested: A comprehensive, standardized data extraction form will be developed using Microsoft Excel and piloted for variable extraction by two independent reviewers (JZ and NFJ). The identified citations and extracted data will be regularly backed up to prevent data loss. Demographic data will include the first author’s name, year of publication, country, study design, and experimental study used to form an initial development. Two reviewers (JZ and NFJ) will pilot the form on a random sample of 20% studies, which representing diverse study designs, that meet the inclusion criteria. The reviewers will refine the form accordingly while identified by any difficulty after discussion. The revised form will be tested on an additional 10% studies to address all concerns. The third independent reviewer (AFML) will provide any additional revision after reviewing the piloted form before finalization. Furthermore, we appreciate your attention to this detailed review about "the data that will be excluded". We would like to revise this section as you suggested: the data that will be included. | Page 9-10, line 238-247, 249. |
| 6. Text :"The quality assessment on the included publications was carried out using the SYRCLE’s risk of bias tool”:  This statement is clear but needs more detail on how each criterion of the SYRCLE tool is applied. Be sure to describe details such as Type of bias, Domain, Description of domain, Review authors judgment for this part either in the text or in the form of a table.  Text: "were the included studies synthesized;"  Explain the methods used to synthesize the data, whether it is a narrative synthesis, meta-analysis, etc. | We appreciate your attention to this important section of our protocol. We would like to revise this section as you suggested: For each domain, the reviewers will make a judgment to the risk of bias as ‘low’, ‘high’, or ‘unclear’. If a study to be included with a ‘low’ risk of bias judgment, at least four criteria must be meet. The process of the quality assessment on included studies will be evaluated by independent reviewers (JZ, NFJ and AFML). To minimize selection bias, we will employ independent screening of titles, abstracts, and full texts. Any disagreements will be resolved through discussion or consultation by a fourth reviewer (NFR). Considering study limitations, incomplete outcome, imprecision, and publication bias, any discrepancies or disagreements, a fourth reviewer (NFR) will be consulted if possible. In addition, part of description has been mentioned in page 11-12, lines 266-280. For the section of ‘Data syntheses has been revised in page 10-11, lines 282-286. | Page 11-12, line 266, 272-286. |
| 7. Expected results |  |  |
| Text: "This systematic review will provide strong evidence for the hypothesis that MALAT1 is involved in the pathophysiological processes of CAD in animal and in vitro studies."  The statement is clear but could be strengthened by specifying the types of evidence expected.  Clarify the types of evidence (e.g., molecular, cellular, physiological) expected to support the hypothesis. | We appreciate your attention to this important section of our protocol. We would like to revise this section as you suggested: This systematic review aims to provide strong evidence supporting the hypothesis that MALAT1 is involved in the pathophysiological processes of CAD in animal and in vitro studies. For animal studies, it will gather molecular, cellular, or physiological evidence on how MALAT1 potentially involved in progression of CAD. Additionally, it will complement existing evidence based on the Klotho/FGF23 axis and Wnt/β-catenin and ERK/MAPK as well as other signaling pathways, as potential molecular targets and vascular/biological changes, such as endothelial cell dysfunction, inflammatory cell activation, and etc. By synthesizing evidence of these studies, this protocol will not only strengthen the hypothesis of lncRNA involved in the pathophysiological of CAD, but also provide a method to overview the literature in the field of CAD and lncRNA biology. | Page 11, line 289-297. |
| 8. Discussion |  |  |
| The discussion provides a broad overview but lacks in-depth analysis of the findings. It should delve deeper into how MALAT1 influences specific pathways and the implications for CAD.  More critical evaluation of the studies referenced is needed. Discuss their methodologies, limitations, and the strength of their findings.  There should be a clearer link between the presented evidence and the initial hypotheses or research questions. Explicitly state how each piece of evidence supports or refutes your hypotheses.  text: "The regulation of MALAT1 intracellular signaling pathways is diverse and highly complex as the same molecule may have different regulatory effects in different models."  This is a broad statement. Give specific examples to illustrate this complexity. | We appreciate your comments regarding the discussion section of our manuscript. We would like to clarify our approach to this section in the context of this study protocol:  (1) We would like to consider that as this manuscript presents a protocol for a systematic review, rather than the results of the review itself, we have intentionally kept the discussion section concise and focused on the potential implications and importance of the proposed study.  (2) At this stage, we do not have detailed evidence to support an in-depth exploration of the specific pathways through which MALAT1 is involved in and affects CAD. Such detailed discussions will be more appropriate and valuable in the final systematic review article, when we have synthesized and analyzed all the relevant studies.  (3) We would like to provide a comprehensive, critical, and in-depth discussion of our findings, including specific pathways and mechanisms, in the final systematic review manuscript. It will including: i) a detailed analysis of how MALAT1 involved in specific pathways in CAD; ii) critical evaluation of the methodologies and limitations of included studies; iii) clear links between the evidence found and our initial hypotheses; iv) specific examples illustrating the complexity of MALAT1 involved in divers models. | Page 13-14, line 299-339. |
| 9. Table1: |  |  |
| Population:  The inclusion criteria for both animal and in vitro studies are comprehensive and well-detailed. However, the clause “given at any time and diet condition” should be more specific to provide clear guidelines on acceptable variations in diet and timing. | We appreciate your comments regarding the table section of our manuscript. We would like to clarify our approach to this section of time and diet. For CAD animal models, we have mentioned the criteria to include the construction of acute and chronic models. However, we will care about the included studies and consider any observed effects of diet during the data extraction and synthesis. We would like to consider this approach contribute to interpretate any dietary influences on the target molecules or pathways. | Page 6, line 142-150;  Supporting Information S1. |
| Comparator:  Exclusion criteria: The exclusion of studies without comparator or control groups is justified, but the specifics around "lnc-MALAT1 in CAD" are restrictive and should be explained to clarify why this particular focus is crucial. | We appreciate your comments regarding this section of our protocol. We aim to evaluate the role and mechanism of lncMALAT1 that may contributed to CAD based on animal and in vitro studies. We would like to consider that with the restrictive of "lnc-MALAT1 in CAD" contribute to directly address the key role of crucial target lncRNA, MALAT1, in the pathophysiology of CAD. | Page 6, line 150-154;  Supporting Information S1. |
|  |  |  |
| **Journal Editor: Seyed Mohammad Gheibihayat** | | |
| **Academic Editor Comments** | | |
| Please use the space provided to explain your answers to the questions above and, if applicable, provide comments about issues authors must address before this protocol can be accepted for publication. You may also include additional comments for the author, including concerns about research or publication ethics.  You may also provide optional suggestions and comments to authors that they might find helpful in planning their study.  (Please upload your review as an attachment if it exceeds 20,000 characters) | Dear Prof Seyed Mohammad Gheibihayat,  Greetings.  We deeply appreciate your vulnerable contributions to our study ***protocol*** article. Thanks for the comments. We would like to revise the article and response each comment very carefully based on the following suggestions and comments from each reviewer to make it more readable. We have described the sufficient detailed methodology, databases, and precise search queries in the protocol article to ensure that all analyses could be replicated by other researchers using the same method. Moreover, we would like to state all data underlying the findings will be made available when the study is complete based on the policy of PLOS ONE. |  |
|  |  |  |
| **R2** | | |
| **Reviewer Comments** | **Response** | **Revised Location in Manuscript (file name: Revised Manuscript with Track Changes)** |
| **Reviewer #3: Sareh Bakhshandeh Bavarsad** | | |
| **Comments:** | | |
| The revised manuscript has addressed all the points raised in the initial review. The improvements made are satisfactory, and the manuscript is now acceptable for publication. | Dear Prof. Sareh Bakhshandeh Bavarsad  Greetings.  We sincerely thanks for your time, effort, and thoughtful consideration in accepting our revised manuscript for publication. We are pleased to hear that our previous revisions have addressed all the concerns raised in the previous review and that the improvements have met your expectations. We greatly appreciate the detailed and comprehensive review process, as well as the constructive feedback provided. |  |
|  |  |  |
| **Reviewer #4:** | | |
| Overall: |  |  |
| Thanks for the opportunity to review this manuscript. I think everything is fine. However, I may have a few concerns: | Dear Prof.,  Greetings.  We deeply appreciate your thorough contribution of our manuscript, and comprehensive review process, as well as the valuable comments provided. Your comments are valuable for further improving the manuscript. We have carefully considered each item and have made the following responses and revisions to address your concerns. |  |
| **Comments:** | | |
| 1. I think putting a section named "results" in the abstract is somehow redundant for a protocol study. Consider removing it. | We appreciate your thoughtful comment regarding the inclusion of a "Results" section in the abstract. We understand the concern that protocol manuscript not typically to present results in the abstract.  Therefore, we agree with your point that the inclusion of this section may be unnecessary and potentially redundant in the context of a protocol manuscript. Next, we removed the “Results” section in the abstract of the revised manuscript, ensuring that it better matches the format and expectations of a protocol study. | Page 2, line 46-49 |
| 2. Please provide full strategy for searching each database as a table in your manuscript. | We fully appreciate your valuable comment regarding the importance of providing a clear and transparent strategy for database searches in a systematic review protocol, as it contributes to ensure reproducibility for any site performing this process.  However, we have included the full search string in the revised manuscript to address your concern, even though the full strategy for searching each database is present in Table 1 and Supporting information S2 of the current manuscript. This detailed full strategy information on the search terms and relevant parameters for each database, ensuring that the methodology is fully transparent and easily replicable. | Page 7, line 188-191 |
| 3. In your PRISMA figure, please state the full precise exclusion reasons for the full-text reviewing stage. | We greatly appreciate your suggestion regarding the PRISMA flow diagram, which has further strengthened our protocol. We recognize the importance of providing clear and precise reasons for exclusions during the full-text review stage, as it enhances the transparency and rigor of the systematic review process.  Moreover, we have updated the PRISMA diagram to include the full and precise reasons for exclusions at the full-text review stage to address your concern. These reasons are clearly mentioned, which may improve the clarity and completeness of the manuscript. | Page 10, Figure 1 PRISMA flow diagram for systematic reviews |
| 4. First paragraph of the introduction is a bit too long. Consider summarising it. | We greatly appreciate your constructive comment. We agree with your point that the first paragraph of the introduction was somewhat lengthy and could benefit from greater conciseness to enhance readability.  To address your concern, we have revised the first paragraph, summarizing the key points more concisely while retaining the essential information. We believe the revision improves the readability of the introduction and ensures a clearer presentation of our objectives. | Page 3-4, line 61-79 |
|  |  |  |
| **Journal Editor** | | |
| **Academic Editor Comments** | | |
| PLOS ONE  Journal Requirements:  Please review your reference list to ensure that it is complete and correct. If you have cited papers that have been retracted, please include the rationale for doing so in the manuscript text, or remove these references and replace them with relevant current references. Any changes to the reference list should be mentioned in the rebuttal letter that accompanies your revised manuscript. If you need to cite a retracted article, indicate the article’s retracted status in the References list and also include a citation and full reference for the retraction notice.  [Note: HTML markup is below. Please do not edit.] | Dear ***PLOS ONE*** Editors,  Greetings.  We appreciate your suggestion regarding the completeness and accuracy of the reference list. After thorough crosscheck for each citation with trusted databases, including PubMed and Web of Science since receiving the Journal's requirement, we have verified that there are none of the retracted articles being cited in our manuscript. Moreover, we have also ensured that all references in our manuscript are relevant to the topic. Therefore, no changes to the reference list are necessary. If there are any further details or adjustments, please feel free to let us know. |  |
|  |  |  |
| **R3** | | |
| **Reviewer Comments** | **Response** | **Revised Location in Manuscript (file name: Revised Manuscript with Track Changes)** |
| **Reviewer #3 Sareh Bakhshandeh Bavarsad** | | |
| Comments: | | |
| The manuscript is **suitable** for publication in PLOS ONE as a protocol study, pending minor revision: Consider adding a section elaborating on planned statistical methods for synthesizing and analyzing data. | Dear Prof. Sareh Bakhshandeh Bavarsad  Greetings.  We deeply appreciate your thorough contribution of our manuscript, and comprehensive review process, as well as the valuable comments provided. Your comments are valuable for further improving the manuscript. We have carefully considered the item and have added the following section of planned statistical methods for responses and revisions to address your concerns. | Page 11, Line 276-286 |
|  |  |  |
| **Reviewer #4** | | |
| Comments: | | |
| Thanks to the authors for their accurate responses on my comments. I think the manuscript is now **suitable** to be accepted | Dear Prof.,  Greetings.  We sincerely appreciate your time, effort, and thoughtful consideration in accepting our revised manuscript for publication. We are pleased to hear that our previous revisions have addressed your concerns. Moreover, we greatly appreciate the detailed and comprehensive review process, as well as the constructive feedback provided. |  |
|  |  |  |
| **Journal Editor: Shaghayegh-Khanmohammadi** | | |
| Academic Editor Comments: | | |
| It seems to be a scoping review rather than a systematic review. | Dear **Prof. Shaghayegh-Khanmohammadi**  Greetings.  We greatly appreciate your time, effort, and thoughtful comments on our revised manuscript. We have carefully considered each item and have made the following responses to address your concerns.  **On the one hand,**  The distinction between a systematic review and a scoping review depends on the objectives, methodologies, and reporting frameworks. Our protocol aligns with the definition, purpose, and methodological rigor of a systematic review, as outlined by the PRISMA-P guideline. Next, we would like to discuss this classification through the following seven key points:  **1. Focus on clear research questions and hypothesis.**  Systematic reviews are characterized by specific, predefined research questions aimed at synthesizing evidence to address a well-defined knowledge gap. In contrast, scoping reviews map broad evidence scopes without prioritizing depth of synthesis or hypothesis testing. Importantly, our protocol explicitly states three research questions:  a. Do lncMALAT1 has roles in CAD?  b. Do lncMALAT1 activating upstream signalling Klotho/FGF23 and regulate the downstream Wnt/β-catenin or ERK/MAPK pathway or any other signaling pathways?  c. What are the potential molecular changes, such as migration, proliferation, lumen formation and apoptosis associated with lncMALAT1?  These questions are hypothesis-driven, seeking to evaluate causal relationships (e.g., "Does lncMALAT1 activate Klotho/FGF23?") and mechanistic outcomes (e.g., apoptosis rates, pathway activity).  Such specificity is different from the exploratory, non-hypothesis-driven nature of scoping reviews.  **2. Adherence to the PRISMA-P and PICOS framework**  Our protocol strictly follows the PRISMA-P checklist and the PICOS framework (Population, Intervention, Comparison, Outcome, Study design), indicators of systematic reviews.  For instance:  a. Population: CAD animal models and hypoxia-induced cardiomyocytes.  b. Intervention: Genetic/cellular/ pharmacological modulation of lncMALAT1.  c. Comparison: Control groups (sham, blank vehicle, healthy).  d. Outcomes: Quantifiable pathway activity (e.g., fold changes in ERK/MAPK expression) and biological changes (e.g., apoptosis rates).  e. Study design: Controlled experimental studies.  In contrast, scoping reviews rarely use PICOS or narrowly defined eligibility criteria. They prioritize broadness over specificity.  **3. Pre-registered protocol with PROSPERO**  Our systematic review protocol was registered on PROSPERO (CRD42024504245), a registry exclusive to systematic reviews. Scoping reviews are not eligible for PROSPERO registration, further validating our classification.  **4. Rigorous data synthesis and quality assessment**  Systematic reviews employ structured methods to synthesize data and assess bias. Our protocol includes:  a. Risk of bias assessment: Using SYRCLE’s tool for animal studies.  b. Data extraction: Quantitative outcomes (e.g., fold changes in gene expression, apoptosis percentages).  c. Narrative synthesis: Tabulated and graphical presentation of pathway activity and biological changes.  Scoping reviews omit bias assessment and rarely extract numerical data, focusing instead on thematic categorization.  **5. Exclusion of non-experimental and grey literature**  We exclude reviews, conference abstracts, and grey literature—a decision aligned with systematic reviews aiming to synthesize primary evidence.  Scoping reviews typically include these sources to map all available evidence.  **6. Focus on explicit primary and secondary outcomes**  Our primary and secondary outcomes are explicitly defined and measurable:  a. Primary: Identification and characterization of Klotho/FGF23 and Wnt/β-catenin and ERK/MAPK pathways for both animal and in vitro studies.  b. Secondary: Other pathways (e.g., PI3K/AKT) and biological changes (e.g., proliferation).  Scoping reviews will not predefined outcomes or prioritize the quantitative indicators.  **7. Aims to elaborate new perspectives and targeted treatments for CAD clinically.**  The aim of our review is to identify therapeutic targets for CAD patients who are intolerant to invasive therapies. This mechanistic evidence-based translational focus is uncommon for scoping reviews that prioritize descriptive knowledge mapping.  **Conclusion**: The structured methodology, hypothesis-driven questions, PROSPERO registration, and plans of quantitative synthesis clearly categorize our protocol as a systematic review.  However, scoping reviews would lack the rigorous process work of specificity, bias assessment, quantitative data synthesis and outcome evaluation presented here. |  |
| Academic Editor additional Comments: | | |
| The manuscript was reassigned to me and I am not the first editor of the manuscript; thus, this manuscript was not assigned to me 10 months ago. The main issue of this protocol is that it includes no numerical values for data synthesis. All the protocols attached to your email investigate at least one numerical value (e.g. frequency, measure of effect, etc.). I think if you consider your protocol as a scoping review, it would be much better. Moreover, some reviewers suggested rejection in the first round of review. However, if you still do not agree with the decision, you can stay in contact with the editorial office (cc email). | **Dear Prof. Shaghayegh-Khanmohammadi**  Greetings.  We greatly appreciate your time, effort, comprehensive review process, and thoughtful comments on reconsidering our revised manuscript. We have carefully considered the item you have mentioned and made the following responses to address your concerns.  Furthermore, we have added a specific improved section in the manuscript that named “**Planned statistical methods**” to describe the process of data synthesis. | Page 11, Line 276-286 |
